# Dual Inoculations of Dark Septate Endophytic and Ericoid Mycorrhizal Fungi Improved the Drought Resistance of Blueberry (*Vaccinium corymbosum* L.) Seedlings

**DOI:** 10.3390/jof12050356

**Published:** 2026-05-12

**Authors:** Xiaolan Guo, Jinbin Hu, Yaqin Wang, Lingda Zeng, Dun Wang, Yu Cao, Delu Wang

**Affiliations:** 1 College of Life Sciences, Huizhou University, Huizhou 516007, China; gxl2022@hzu.edu.cn (X.G.); 2 College of Forestry, Guizhou University, Guiyang Huaxi 550025, China; 3 School of Architecture and Civil Engineering, Huizhou University, Huizhou 516007, China

**Keywords:** mixed inoculation, resilience, physiological characteristics, blueberry

## Abstract

Dark septate endophytes (DSE) and ericoid mycorrhizal fungi (ERMF) are employed to augment the abiotic stress resistance of fruits. However, their potential functions in enhancing the drought resistance of blueberry, an economically important fruit, remain unclear. Thus, this study aims to identify optimal inoculation combinations to enhance the drought resistance of blueberry seedlings. Specifically, the effects of single and dual inoculations with DSE (*Cladosporium cladosporioides*, D79) and ERMF (*Oidiodendron citrinum*, N12) on seedling physiology and metabolism were explored under varying drought conditions. The results showed that dual inoculation significantly improved leaf physiological characteristics. Under severe drought stress, the 1:2 DSE:ERMF ratio (D1N2) notably increased leaf relative water content (RWC) and reduced electrolyte leakage by up to 42.1% compared with the non-inoculated control. Dual inoculation also significantly decreased malondialdehyde (MDA) content, with the smallest increase observed in D1N2. Regarding antioxidant enzymes, dual inoculation sustained higher superoxide dismutase (SOD) activity under moderate drought and minimized the decline in SOD activity under severe drought (the lowest decrease was 36.4% in D1N2 versus 56.7% in CK). Moreover, the antioxidant losses under drought stress were reduced by upregulating various metabolic processes, especially the biosynthesis of phenylalanine, tyrosine, and tryptophan. A comprehensive evaluation suggested that inoculation with a 1:2 mixture of DSE and ERMF most effectively improved blueberry drought resistance, primarily by enhancing water and metabolite supply and stimulating the antioxidant defenses.

## 1. Introduction

Drought is among the most critical environmental stresses limiting global plant production, mainly by hindering plant growth and metabolism [1,2]. Its significant constraints on regulatory enzymes and metabolites limit plant growth and crop yields [3,4]. Characteristics of plants under drought stress include wilting, stomatal closure, reduced osmotic and water potential, lowered water use efficiency, and decreased relative water content (RWC) in leaves [5]. Reactive oxygen species (ROS), including superoxide, hydroxyl, and hydrogen peroxide radicals, are also produced under drought stress [6]. By reacting with various cellular components, ROS damages membranes and other crucial macromolecules, such as proteins, nucleic acids, lipids, and photosynthetic pigments (chlorophyll and carotenoids) [7].

The symbiosis between endophytic fungi and host plants has recently attracted much attention as an indirect means of mitigating environmental change. Host plants provide nutrients for endophytic fungi, while the latter adjust the hormone levels of the former, modifying their growth and enhancing their tolerance to harsh environments [7,8]. Thus, suitable fungal strains can effectively improve plant productivity and ecological adaptability.

Dark septate endophytes (DSE) are ascomycetes commonly colonizing the roots of both mycorrhizal and non-mycorrhizal plants. Most plants form complex and varied symbiotic relationships with fungi, which contribute to their adaptation to drought stress [9,10]. According to He et al. [10], DSE inoculation promoted the growth and fruit ingredient content of *Lycium ruthenicum*. Recent studies have further confirmed that DSE inoculation can significantly enhance drought tolerance in various plants, including blueberry (*V. corymbosum*), by modulating phytohormones, improving antioxidant capacity, and regulating non-structural carbohydrates [11,12]. For instance, a novel growth-promoting DSE fungus improved drought tolerance in blueberry seedlings through physiological adjustments and hormone signaling [11]. In addition, DSE have shown potential in promoting growth and drought resistance in other species such as desert plants and turfgrasses under water deficit conditions [13,14]. Various DSE species and inoculum levels affect plants differently. For example, Li et al. [15] found that inoculations with different DSE species exerted varied effects on the growth of *Artemisia oleifera* under drought stress. Zuo et al. [16] found through inoculation experiments with varying doses that two DSE species symbiotized with *Hedysarum scoparium* and promoted its growth, and that the effects varied by DSE species and inoculum level, with an optimal dose of 20 mL. More importantly, these effects were generally beneficial. Therefore, different DSE species and doses may have different effects on plant stress resistance.

Ericaceae is a large family of small plants and shrubs commonly grown in infertile acidic soils [17], which symbiotize with ericoid mycorrhizal fungi (ERMF). While the taxonomic status of ERMF has received more attention than ectomycorrhizal and arbuscular mycorrhizal associations, little is known about their functional significance and responses of ERMF associations to various environmental conditions [18,19]. The effectiveness of ERMF in enhancing the stress resistance of ericaceous plants is particularly unclear. Mu et al. [20] found that lowland plants benefited more from mycorrhizal colonization than upland plants, exhibiting greater improvements in plant growth and drought tolerance. However, the non-inoculated, well-watered upland plants showed greater dry weights than the non-inoculated lowland plants after three weeks of treatment. They hypothesized that these differences were offset since ERMF inoculation significantly improved the drought resistance of both upland and lowland plants. A previous study reported that *Oidiodendron maius* and *Meliniomyces variabilis* improved the salt tolerance of three ericaceous species, and their effects on growth and physiological parameters varied with fungal and plant species [21].

With shallow roots and lacking root hair, blueberry plants are intolerant to drought [22]. In southern China, blueberries are mainly cultivated in hilly and mountainous areas with poor water availability and low water retention capacity, with frequent local or intermittent droughts. Although scholars confirmed microbes can improve blueberry performance under drought stress [20], existing studies have mainly focused on single inoculation with beneficial fungi [15,23,24]. Mixed inoculation provides stronger stress resistance than a single inoculation. Currently, symbionts based on single inoculations with DSE and ERMF at different doses have shown better responses to drought stress, but the response mechanism of dual inoculation has not been reported. Thus, this study investigates the effects of their dual inoculations on the growth, leaf physiological characteristics, and secondary metabolites of blueberry (*V. corymbosum*) seedlings under drought stress. The following hypotheses are proposed:

(i) Inoculation with DSE and ERMF increases the drought stress resistance of blueberry seedlings by positively influencing physiological traits and secondary metabolite production.

(ii) Optimal inoculation ratios can significantly mitigate the adverse effects of drought stress on blueberry seedlings under severe water deficit.

## 2. Materials and Methods

### 2.1. Test Materials and Design

#### 2.1.1. Test Site

The experiment was performed in September 2019 in the laboratory and nursery of the Forestry College of Guizhou University (26°25′ N, 106°40′ E), located in Huaxi District, Guiyang City, Guizhou Province. The study area featured a humid subtropical monsoon climate, with an average annual temperature of 15.6 °C (23.3 °C in summer and 6.7 °C in winter). Its maximum and minimum temperatures are 33.4 °C and −3.8 °C. Its annual precipitation is 1450.8 mm, and its annual relative humidity is 79%. Its annual light duration is 1287.4 h, and the annual frost-free period spans 352 days (Guo et al., 2023) [25].

#### 2.1.2. Test Strains

The DSE group included *Phialocephala* sp. (D05), *Rhizopycnis vagum* (D20), *Phialocephala fortinii* (D65), *Cladosporium cladosporioides* (D79), and *Cladosporium* sp. (D37). The ERMF group included *Oidiodendron citrinum* (N12), *Trametes versicolor* (N40), *Helotiales* sp. (N17), *Thozetella* sp. (N89), and *Acephala* sp. (N07) (Table 1).

#### 2.1.3. Test Medium and Strain Screening

DSE and ERMF strains were activated on standard PDA medium (comprising 200 g potato, 20 g glucose, 20 g agar, and 1000 mL distilled water). Drought stress was simulated using PDA overlaid with polyethylene glycol (PEG)-6000 at final concentrations of 0%, 5%, 10%, 15%, and 20% (*w*/*v*), corresponding to osmotic potentials of 0, −0.1, −0.3, −0.53, and −0.79 MPa, respectively [27].

Briefly, PEG-6000 (0, 50, 100, 150, or 200 g) was dissolved in 1000 mL potato dextrose broth (without agar) supplemented with 2 mmol/L MES. Then, the mixture was adjusted to pH 5.8 ± 0.1 and autoclaved (121 °C, 20 min). Sterile PDA plates were prepared separately. On a clean bench, equal volumes of cooled PEG-containing liquid media were poured onto solidified PDA plates and allowed to equilibrate for 3 days. Excess liquid was then discarded, yielding PEG-infused solid media ready for subsequent use.

The strains with strong drought resistance were screened as follows: the tested fungi were activated on PDA plate media and cultured at 28 °C for 3 days. As the strains reached the vigorous growth stage and were 1 cm from the edge of the Petri dish, they were inoculated onto solid media containing different concentrations of PEG-6000 and cultured inversely at 28 °C for two weeks. Mycelia growth was monitored, and colony diameter was measured and recorded. The ERMF and DSE strains exhibiting strong drought resistance were screened.

The low-inhibitory strain combinations were screened as follows: the selected ERMF and DSE strains were tested against each other. According to the plate confrontation method proposed by Hussein et al. [28], the ERMF cake (diameter 5 mm) was attached to the PDA plate, and the DSE cake (diameter 5 mm) was inoculated on the opposite side, with a distance of about 5 cm between them. The fungi were incubated at 28 °C for 3 days. Each group was triplicated, and each replicate involved 2 plates. The colony diameters with single-strain inoculations served as the control. The antagonistic effect between the two strains was preliminarily determined based on the diameters of the antagonistic and control strains. A greater distance indicates a stronger antagonistic effect between the two strains. The radius of the two colonies pointing to the center of the plate was measured using the crossover method, and the inhibition rate was calculated using the formula proposed by Chen et al. [29]:

(1) $$\begin{array}{c} I=\frac{d_{i}-d}{d_{i}}\times 100\% \end{array}$$

where *I* denotes the inhibition rate; *d_i_* represents the colony diameter when strain *i* is cultured alone; and *d* represents the colony diameter when the strain is cultured in confrontation with other strains. The inhibition rate of the face-off combination is the sum of the inhibition rate of two face-off strains and a single cultured strain. The strain combination with the lowest inhibition rate was selected as the sample for the inoculation test.

The fungal suspension was prepared as follows: potato dextrose broth, sterilized at 121 °C for 30 min on the sterile operating table, was added to a 250 mL conical flask (roughly 200 mL per bottle) and sterilized at 121 °C for 45 min. After cooling, the activated fungal strains were placed into the bottle (8 mm) and incubated for 14 days on a constant temperature shaker at a speed of 150 r/min and a temperature of 25 °C.

#### 2.1.4. Drought Stress Experiment

PEG-6000 was used to simulate drought stress, and D79 and N12 were screened for dual inoculation experiments. In June 2020, 6-month-old blueberries (*V. corymbosum*. ‘O’Neal’) were inoculated using the root irrigation method (pouring fungal solution evenly into holes punched around the rhizosphere and filling the holes). Infection tests were conducted on all plants after inoculation to confirm successful infection [25].

The cultivation substrate consisted of peat soil, pine needle humus, and perlite in a ratio of 1.5:2:1. After sterilizing at 0.1 MPa and 121 °C for 2 h, the substrate was dried in natural air. Sulfur powder was added to adjust the pH to 4.7 for later use. The final substrate had a bulk density of 0.75 N/m^3^, an aeration porosity of 13.9%, a pH of 4.7, an organic carbon content of 14.76 g/kg, a total nitrogen of 0.70 g/kg, a total phosphorus of 0.10 g/kg, a total potassium of 0.5 g/kg, an available nitrogen of 84.93 mg/kg, an available phosphorus of 98.14 mg/kg, and an available potassium of 102.18 mg/kg. The sterilized and cooled substrate was filled into plastic nursery pots (15 cm upper diameter × 10.5 cm lower diameter × 11.5 cm height) that had been disinfected with potassium permanganate. After filling the substrate to two-thirds of the pot’s height, healthy, uniform-sized seedlings free from pests and diseases were transplanted into the pots for establishment. The seedlings were acclimatized for two months before transplantation. Each treatment was triplicated, with eight pots per replicate.

For each pot, 21 mL of fungi solution was inoculated. The mixed treatments were established with different volumetric ratios (Table 2). The six treatments set up included no inoculation (control, CK), single DSE inoculation (D), single ERMF inoculation (N), dual inoculation with DSE and ERMF at a 1:1 ratio (D1N1), dual inoculation with DSE and ERMF in a 1:2 ratio (D1N2), and dual inoculation with DSE and ERMF in a 2:1 ratio (D2N1). Samples were collected two months after inoculation to determine relevant growth, physiological, and biochemical indicators.

The six treatments were subjected to four soil water content treatments: 70% to 80% (normal water content, T1), 55% to 65% (mild drought, T2), 35% to 45% (moderate drought, T3), and 15% to 25% (severe drought, T4). Each soil water content treatment was triplicated, with three pots per replicate (216 pots in total). According to the method proposed by Yang et al. [30], the total control weight of potted seedlings in treatment groups T1, T2, T3, and T4 can be calculated as follows:

(2) $$\begin{array}{c} TW=BW+PW+\frac{SW}{1+SC}\times \left(1+RWC\times FC\right) \end{array}$$

where *TW* represents the total weight; *BW* denotes the basin weight; *PW* is the plant weight; *SW* indicates the soil weight; *SC* represents the soil water content; *RWC* is the relative soil water content, which is the percentage of soil water content in the field water holding capacity of the soil; and *FC* denotes the field water holding capacity. The control weights under the four water content levels of each treatment were: T1: 0.24 kg–0.27 kg, T2: 0.20 kg–0.23 kg, T3: 0.14 kg–0.17 kg, and T4: 0.08 kg–0.11 kg. The pots were weighed at 5 p.m. every two days to compensate for the water loss during the day. After 30 days of drought, samples were collected to determine relevant physiological indicators.

### 2.2. Indicator Determination

The following indicators were determined using 1 g of leaf tissue. Chlorophyll content was determined after extraction with an 80% acetone solution, and the soluble protein content was determined using the Coomassie brilliant blue-G250 staining method [31]. The anthrone method (colorimetry) was used to determine soluble sugars, and the nitro blue tetrazolium photochemical reduction method was used to determine superoxide dismutase (SOD) activity [32]. Malondialdehyde (MDA) activity was determined using the thiobarbituric acid method [33]. The proline (Pro) content was determined by the acidic ninhydrin chromogenic method, where the relative conductivity of the leaves was measured with a conductivity meter, and their water potential was determined using the pressure chamber method. The metabolite component analysis was based on non-targeted metabolomic assays. Metabolite profiling was performed using a widely targeted metabolome method and assay kits from Lianchuan Biotechnology Co., Ltd. (Hangzhou, China). Specifically, leaves were sampled from different drought stress treatments, and freeze-dried leaves were crushed into powder in a mixer mill (MM 400, Retsch, Hangzhou, China). A total of 100 mg powder was extracted overnight at 4 °C with 1.0 mL 70% aqueous methanol before centrifuging at 10,000× *g* for 10 min. After that, the extracts were absorbed, filtered, and analyzed by an LC-MS/MS system. The Kyoto Encyclopedia of Genes and Genomes (KEGG) enrichment analysis is depicted in D1N2_VS._CK differential metabolites, and the screening conditions are *p* < 0.001 among the top 20 (Supplementary Table S1). The metabolite differences between treatment D1N2 and CK were analyzed.

### 2.3. Data Analysis

#### 2.3.1. Statistical Analysis

The data were processed in Microsoft Excel 2010, SPSS 25 (IBM Corp., Armonk, NY, USA), and Origin 2018. Normality of the data was assessed using the Shapiro–Wilk test (*p* > 0.05), and no data transformation was required. Subsequently, the experimental data were subjected to one-way analysis of variance (ANOVA) and Tukey’s significance test. All data are expressed as the mean ± standard deviation with three replicates.

#### 2.3.2. Membership Function Evaluation Method

A comprehensive evaluation was conducted using the membership function evaluation method based on fuzzy mathematics. Specifically, the membership function in a fuzzy control system converts the ordinary, clear quantities into fuzzy quantities for fuzzy logic operations and reasoning. Thus, plant characteristics were comprehensively evaluated based on multiple index determinations. The formulas for the membership function method are as follows:

(3) $$\begin{array}{c} U=\frac{X-X_{min}}{X_{max}-X_{min}} \end{array}$$

(4) $$\begin{array}{c} U=1-\frac{X-X_{min}}{X_{max}-X_{min}} \end{array}$$

where *U* represents the value of the drought resistance membership function for each index; *X* is the measured value; and *X*_max_ and *X_min_* represent the maximum and minimum values, respectively. The membership function and average values were calculated, with a higher average value indicating better treatment. Equation (3) is used if the indicator is positively correlated with drought resistance, and Equation (4) is used if the indicator is negatively correlated with drought resistance [34].

## 3. Results

### 3.1. Screening of Drought-Resistant Strains

#### 3.1.1. Effects of Different Drought Gradients on Fungal Growth

**Figure 1:**
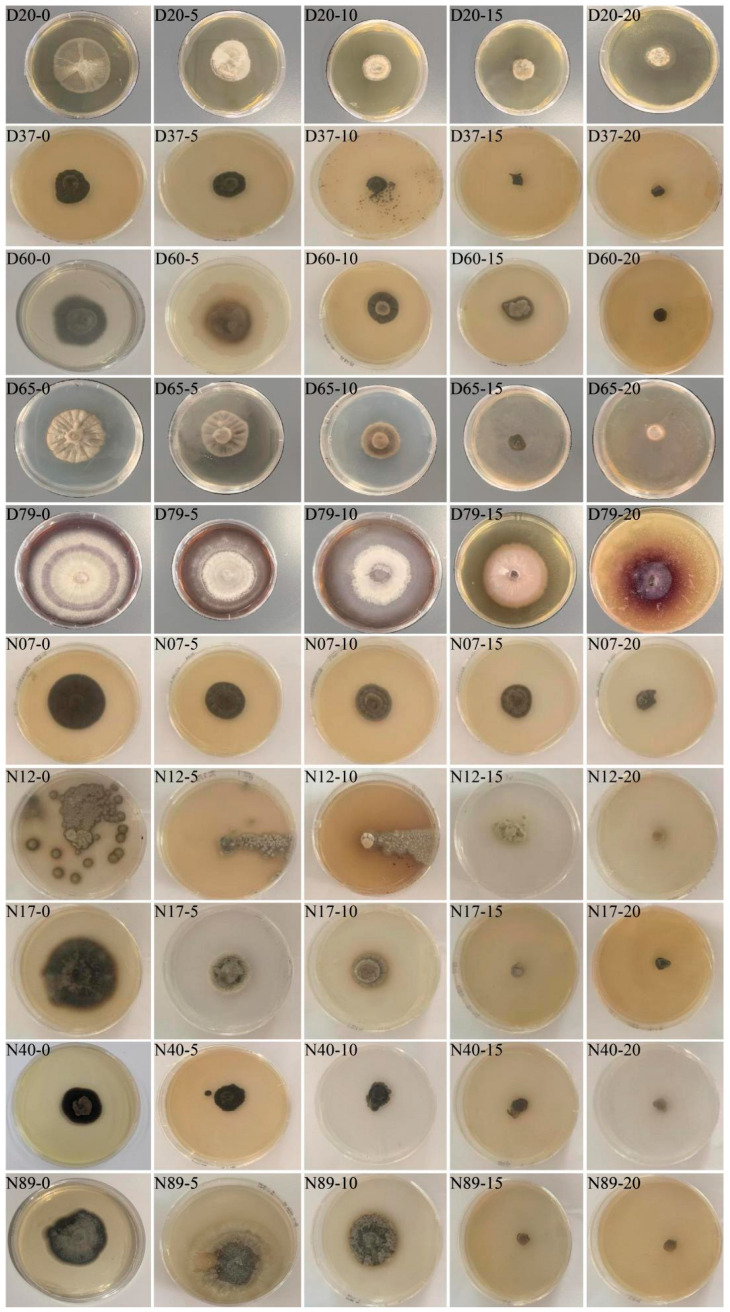
Colonies of ERMF and DSE under different PEG-6000 concentrations.

DSE and ERMF showed different growth characteristics under different drought stress levels. Their growth was significantly affected by the drought stress simulated with PEG-6000 (Table 3). As the stress intensity increased and the osmotic potential decreased, the colony diameters of D79, N12, and N40 exhibited a fluctuating trend of “decrease-increase-decrease,” while the other strains showed a continuous decline. The growth diameters of N12, N07, and N89 under 5% PEG-6000 treatment showed no significant difference compared with the 0% concentration (*p* > 0.05). Notably, the growth rate of N12 did not begin to decline until the stress concentration reached 15%, indicating that a high osmotic environment exerts an inhibitory effect on strain growth. Morphological observations revealed that the colonies of D79 displayed radial grooves. As the osmotic potential decreased, the colony color gradually became lighter and the hyphae became thinner, suggesting that hyphal growth was inhibited. In contrast, the hyphal color of N07 and D37 remained largely unchanged under low osmotic potential. However, for D37, N17, and N12, as the osmotic potential decreased, the hyphal color gradually became lighter, the hyphae became finer, and growth slowed down (Figure 1).

#### 3.1.2. Analysis of the Confrontation Between ERMF and DSE

**Figure 2:**
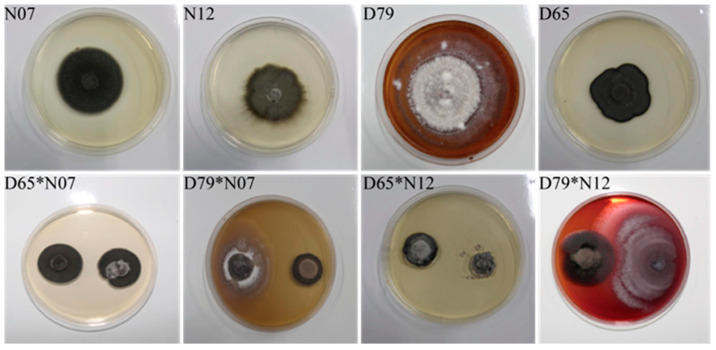
Confrontation situation of ERMF and DSE strains.

The selected DSE and ERMF strains with strong drought resistance were subjected to face-off experiments (Figure 2). *C. cladosporioides* (D79) and *Acephala* sp. (N07) exhibited significant inhibitory effects, while the inhibitory rate of *C. cladosporioides* (D79) and *O. citrinum* (N12) was the lowest. The colony diameters of D79 and N12 showed no significant difference, suggesting their suitability for co-culture (Table 4).

### 3.2. Post-Inoculation Leaf Relative Water Content (RWC) and Electrolyte Conductivity Response Characteristics of Blueberry Leaves Under Drought Stress

**Figure 3:**
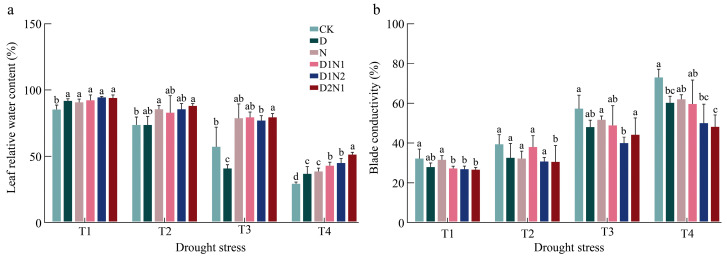
Responses of Leaf relative water content (RWC) and electrolyte conductivity in leaves of inoculated blueberry seedlings to drought. Note: Different lowercase letters indicate significant differences (*p* < 0.05) among inoculation treatments under the same drought intensities; T1: Normal moisture, T2: Mild stress, T3: Moderate stress, T4: Severe stress. (same below). (**a**) shows the Leaf relative water content (RWC) under drought stress under different inoculation treatments; (**b**) shows the electrolyte conductivity under drought stress under different inoculation treatments.

As shown in Figure 3a, leaf RWC across the six inoculation treatments showed a decreasing trend with intensifying drought stress, all of which were minimized under severe drought. Under T2, no significant difference was observed in the leaf RWC among the treatments, indicating that mild drought had little effect on mycorrhizated seedlings. Under T3 and T4, both single inoculation and mixed inoculation increased the leaf RWC, while D2N1 exhibited the most significant effect (Figure 3a).

Under T1, the leaf electrolyte conductivity under mixed inoculation treatments D2N1, D1N2, and D1N1 was relatively low. Under T3, the electrolyte conductivity in each treatment increased rapidly to different extents. Under T4, the phase-pair conductivity of leaves under CK was the highest, which was significantly different from that of D, D1N2, and D2N1, representing increases by 21.1%, 42.1%, and 51.85%, respectively (Figure 3b).

### 3.3. Effects of Inoculation Treatments on MDA and SOD in Blueberry Seedling Leaves Under Drought Stress

**Figure 4:**
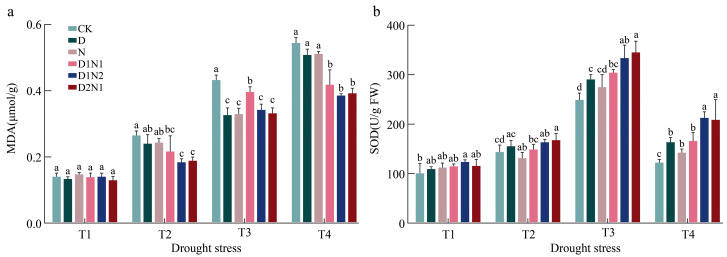
Effects of inoculation treatments on MDA and SOD of blueberry leaves under drought stress. MDA: Malondialdehyde, SOD: SuperOxideDismutase. (**a**) shows the Leaf MDA under drought stress under different inoculation treatments; (**b**) shows SOD under drought stress under different inoculation treatments. The lowercase letters in the figure represent significant differences among different treatments.

The MDA content of blueberry leaves increased continuously with intensifying drought stress. Under T2, the MDA content increased across all treatments (the largest increase was 89% in CK, and the smallest increase was 30% in D1N2). Under T3, MDA started to rise rapidly in all treatments (the largest increase was 199.7% in CK, and the smallest increase was 124% in N). Under T4, the MDA content of CK was significantly higher than that of the other treatments, with increases ranging from 22% to 29% (Figure 4a).

Leaf SOD activity tended to increase first and then decrease with the intensifying drought stress. Under T1, SOD activity in the D1N2 treatment increased significantly by 23.6% compared with CK, while those in the other treatments exhibited no significant difference from CK. Under T2, the leaf SOD activity of D2N1 was the highest, 17.5% higher than that of CK. Under T3, the leaf SOD activity in the six inoculation treatments peaked, and the dual inoculation of D1N2 and D2N1 led to significantly higher activity than that of single inoculation. Under T4, the SOD activity decreased rapidly compared with that under T3 (the largest decrease was 56.7% in CK, and the lowest was 36.4% in D1N2 (Figure 4b)).

### 3.4. Post-Inoculation Response Characteristics of Blueberry Leaf Osmotic Regulators to Drought Stress

**Figure 5:**
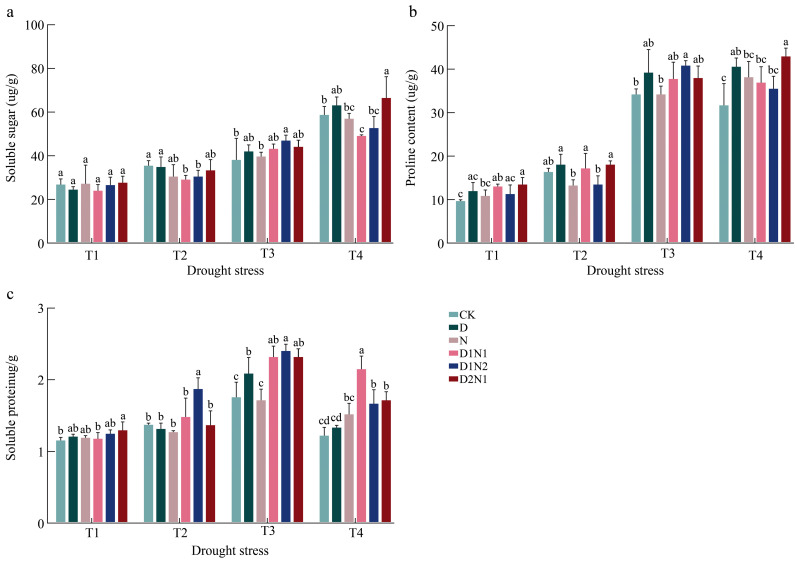
Effects of inoculation treatments on osmotic regulators in blueberry leaves under drought stress. (**a**) shows the Leaf soluble sugar under drought stress under different inoculation treatments; (**b**) shows leaf Proline under drought stress under different inoculation treatments; (**c**) shows leaf soluble protein under drought stress under different inoculation treatments. The lowercase letters in the figure represent significant differences among different treatments.

As drought stress intensified, the soluble sugar content in blueberry leaves increased. Under T1, no significant difference was observed in the leaf soluble sugar content among the treatments. Under T2, the leaf soluble sugar contents of CK, D, and D2N1 increased significantly by 33%, 41%, and 20.7%, respectively, compared to T1, while the remaining treatments showed no significant difference. Under T3, the soluble sugar content increased rapidly across the treatments, with the largest increases observed in D1N2 and D1N1 (54.4% and 48.37%, respectively). Under T4, the soluble sugar content continued to increase and peaked (Figure 5a).

With intensifying drought stress, the Proline content of the blueberry leaves gradually increased. Under T1, the leaf Pro contents of D1N1 and D2N1 reached significantly higher than CK by 32.7% and 37.5%, respectively, whereas the Pro contents of the other treatments exhibited no significant difference. Under T2, the Pro contents of CK, D, and D2N1 increased significantly by 66.7%, 49.7%, and 33.7%, respectively, compared with T1, while those of the other treatments showed no significant increase. Under T3, the free Pro contents of CK, D1N1, and D1N2 increased rapidly and peaked. Specifically, that of D1N2 peaked at 40 μg·g^−1^, 1.2 and 1.19 times that of CK and N, respectively, but was not significantly different from those of the other treatments. Under T4, the free Pro content of CK, D1N1, and D1N2 decreased (Figure 5b).

Under T1, the D2N1 treatment showed the highest soluble protein content, representing a significant increase of 11% compared to CK. Under T2, the soluble protein content of D1N2 was the highest. The leaf soluble protein content peaked under T3. The soluble protein content showed a decreasing trend in all treatments under T4 (Figure 5c).

### 3.5. Comprehensive Drought Resistance Score of Blueberry Seedlings

As shown in Table 5, all treatments had the highest synthesis rate under T3 stress. The comprehensive evaluation scores were all positive (up to 0.751 in D1N2), indicating that all treatments can survive well under T3, where the stress resistance was slightly stronger. Inoculation with DSE and ERMF at a 1:2 ratio achieved the best effect. The membership mean in D1N2 was the highest, followed by D1N1 and N, while that in D was the lowest.

### 3.6. Mycorrhizal Fungi Regulate Secondary Metabolic Pathways to Improve the Drought Resistance of Blueberry Seedlings

**Figure 6:**
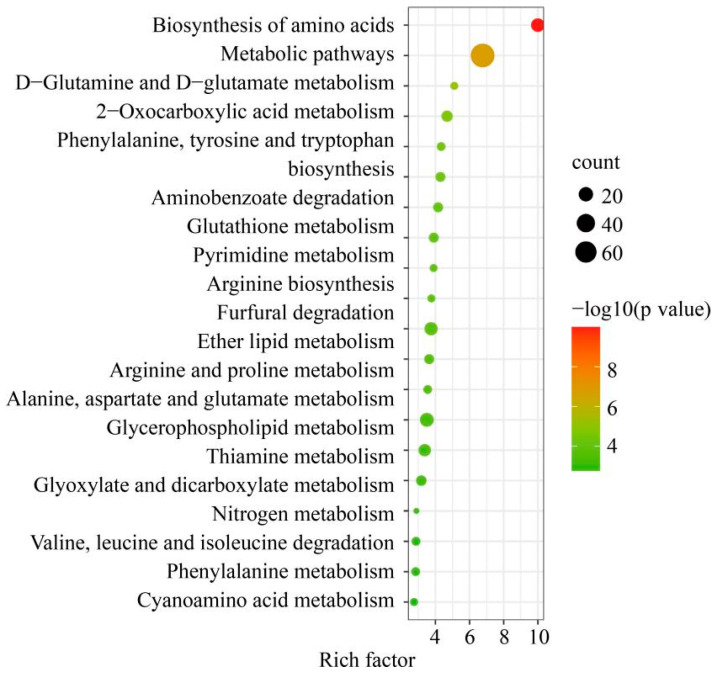
KEGG enrichment analysis of differential metabolites treated with D1N2 and CK under drought stress.

The inoculation treatments significantly enhanced the biosynthesis of amino acids (e.g., phenylalanine, tyrosine, and tryptophan) and various metabolic pathways (e.g., D-glutamine and D-glutamate, 2-oxocarboxylic acid, and nitrogen). These metabolisms are closely related to plant stress tolerance (Figure 6).

## 4. Discussion

The present study provides clear evidence corroborating both hypotheses proposed in the Introduction. Hypothesis (i) posited that inoculation with DSE and ERMF increases the drought stress resistance of blueberry seedlings by positively influencing physiological traits and secondary metabolite production. This was strongly supported. Hypothesis (ii) stated that optimal inoculation ratios can significantly mitigate the adverse effects of drought stress on blueberry seedlings under severe water deficit. This was also corroborated, with the 1:2 DSE:ERMF ratio (D1N2) showing the best performance according to the comprehensive membership function evaluation. These findings are consistent with recent studies highlighting the roles of DSE and ERMF in modulating stress-responsive metabolism and drought resilience in blueberry and other plants [12,14,35].

Drought stress profoundly impacts plant water status, reducing RWC and leaf water potential, which are direct indicators of hydration and wilting severity [23]. Higher RWC indicates enhanced water retention and rehydration, which mitigate cellular damage under water deficit [36]. In this study, dual inoculation with DSE (*Cladosporium cladosporioides*, D79) and ERMF (*Oidiodendron citrinum*, N12) significantly increased RWC in blueberry (*Vaccinium* spp.) seedlings compared to single inoculations and control, particularly under severe drought (15% to 25% field capacity). These results align with the findings of Mu et al. [20] that ERMF inoculation enhanced RWC and water potential in *Vaccinium myrtilloides*, improving the hydraulic conductivity. The superior performance of dual inoculations likely stems from synergistic effects. Specifically, DSE extends extraradical hyphae to enhance soil water uptake, while ERMF improves root hydraulic properties and nutrient acquisition [37]. This synergy likely expands the root-soil interface, which increases water absorption efficiency and maintains cellular turgor, thereby supporting sustained physiological activities under drought stress.

Drought-induced oxidative stress disrupts ROS homeostasis, leading to lipid peroxidation and membrane damage [38]. As a critical antioxidant enzyme, SOD mitigates ROS accumulation [36]. Our results revealed significantly elevated SOD activity under dual DSE-ERMF inoculation compared to single inoculations and CK, indicating enhanced ROS scavenging capacity. These results are consistent with the findings of He et al. [10], who observed increased SOD activity, along with elevated GSH content, soluble protein, and proline in *Lycium ruthenicum* seedlings inoculated with dark septate endophytes under drought stress. Additionally, the MDA level is a marker of lipid peroxidation, and the significantly lower MDA in dual-inoculated seedlings, corroborated by reduced electrolyte leakage, suggests greater membrane integrity. These findings align with the conclusions of Zhang et al. [39] and Chiappero et al. [40], who reported reduced oxidative damage in mycorrhizal plants under drought. The enhanced antioxidant defense in dual-inoculated blueberries likely results from upregulated antioxidant enzymes, as observed in mycorrhizal Sorghum bicolor [41], where fungal symbiosis modulated stress-responsive pathways.

Osmotic adjustment is a key drought adaptation mechanism that involves the accumulation of osmolytes (e.g., Pro, soluble proteins, and soluble sugars) to lower cellular osmotic potential and facilitate water uptake [7]. In this study, dual inoculation markedly increased osmolyte concentrations compared to single inoculations and CK, particularly under severe drought stress. These results corroborate the findings of Xiong et al. [42] that fungal inoculation accelerated osmolyte accumulation and reduced cellular osmotic potential. In particular, Pro not only acts as an osmoprotectant but also stabilizes proteins and membranes under drought stress [43]. The higher osmolyte levels in dual-inoculated seedlings likely reflect enhanced metabolic regulation, possibly mediated by fungal elicitors that upregulate osmolyte biosynthesis genes, as seen in mycorrhizal wheat (*Triticum aestivum*) [44]. This osmotic adjustment likely contributed to the maintained turgor and metabolic activity observed, underscoring the role of DSE-ERMF symbiosis in drought tolerance.

Beyond altering physiological responses, DSE and ERMF also modulate secondary metabolic pathways to bolster drought resistance. Transcriptomic and proteomic analyses revealed enriched pathways in mycorrhizal plants under drought stress, including phenylpropanoid biosynthesis, flavonoid production, and carbohydrate metabolism [41,44]. The dual inoculation in this study significantly increased metabolite abundance, such as phenylalanine, tyrosine, and tryptophan, which are key precursors to phenylpropanoids and flavonoids. The antioxidant properties of flavonoids and their involvement in plant-microbe signaling likely enhance symbiotic efficiency and stress tolerance [45]. These metabolic shifts may be driven by fungal-induced changes in hormone signaling (e.g., abscisic acid and jasmonic acid), which regulate stress-responsive genes, as reported in mycorrhizal *Medicago truncatula* [46]. The enrichment of these pathways in dual-inoculated blueberries suggests a complex interplay between primary and secondary metabolism, which optimizes resource allocation under water scarcity.

Ecologically, the DSE-ERMF synergy highlights their potential as bioinoculants for improving blueberry cultivation in drought-prone regions. However, their varied effects, as seen in differing outcomes across species and inoculum levels [16,47], underscore the need for strain-specific optimization. Moreover, the molecular mechanisms underlying the effects of DSE-ERMF interactions remain underexplored. Future research should leverage multi-omics approaches to elucidate fungal signaling pathways and their regulation of host stress responses. Additionally, field studies are needed to assess the persistence of these benefits in natural settings, where microbial competition and environmental heterogeneity may modulate symbiotic outcomes.

In conclusion, this study demonstrates that dual inoculation with dark septate endophyte (*C. cladosporioides* D79) and ericoid mycorrhizal fungus (*O. citrinum* N12), particularly at a 1:2 ratio, significantly improves the drought resistance of blueberry seedlings. The combined inoculation enhanced leaf relative water content, reduced membrane damage (lower MDA and electrolyte leakage), maintained higher antioxidant enzyme (SOD) activity, promoted osmolyte accumulation, and upregulated key secondary metabolic pathways (especially phenylalanine, tyrosine, and tryptophan biosynthesis). These physiological and metabolic adjustments collectively enabled better water retention, ROS scavenging, osmotic adjustment, and stress tolerance under moderate to severe drought conditions. The 1:2 DSE:ERMF ratio proved most effective according to the comprehensive membership function evaluation. These findings confirm the synergistic benefits of mixed DSE and ERMF inoculation over single inoculation and provide a promising microbial strategy for enhancing blueberry cultivation in drought-prone regions of southern China.

## Figures and Tables

**Table 1:** Inoculation strain information.

Strain No.	Strain Name	Genebank No.	Similarity Index%
N07	*Acephala* sp.	HQ889709.1	99
N12	*Oidiodendron citrinum*	MH864651.1	100
N17	*Helotiales* sp.	KY654693.1	100
N40	*Trametes versicolor*	KY628331.1	100
N89	*Thozetella* sp.	KU059907.1	99.61
D05	*Phialocephala* sp.	MK808244.1	99.42
D20	*Rhizopycnis vagum.*	HQ610503.1	100
D37	*Cladosporium* sp.	MH509413.1	100
D65	*Phialocephala fortinii*	KX440167.1	100
D79	*Cladosporium cladosporioides*	MF077224.1	100

Note: All test strains were isolated and identified from blueberry roots [26].

**Table 2:** Dosages for single and mixed inoculations.

	Treatment	CK	D	N	D1N1	D1N2	D2N1
Dosage/mL							
D79		0	21	0	10.5	7	14
N12		0	0	21	10.5	14	7

Note: D denotes *C. cladosporioides*(D79), and N denotes *O. citrinum*(N12).

**Table 3:** Growth of DSE and ERMF under drought stress.

Strain	The Growth Diameter of Each Strain Under Different PEG Concentrations/cm				
	0%	5%	10%	15%	20%
D05	3.13 ± 0.03 a	2.50 ± 0.06 b	2.07 ± 0.09 c	1.27 ± 0.03 d	0.53 ± 0.03 e
D20	5.13 ± 0.58 a	3.30 ± 0.25 b	2.60 ± 0.15 b	2.17 ± 0.20 bc	1.47 ± 0.32 c
D37	2.73 ± 0.17 a	1.90 ± 0.06 b	2.00 ± 0.28 b	1.35 ± 0.03 c	0.55 ± 0.03 d
D65	5.27 ± 0.09 a	4.13 ± 0.12 b	3.70 ± 0.06 c	3.50 ± 0.06 c	1.40 ± 0.06 d
D79	5.27 ± 0.33 a	4.20 ± 0.06 b	3.50 ± 0.29 bc	3.93 ± 0.35 b	3.13 ± 0.03 c
N07	4.57 ± 0.23 a	4.20 ± 0.15 a	3.07 ± 0.52 b	2.80 ± 0.20 b	1.70 ± 0.21 c
N12	4.63 ± 0.20 a	4.50 ± 0.17 a	5.57 ± 1.02 a	1.80 ± 0.25 b	0.83 ± 0.09 b
N17	3.87 ± 0.18 a	3.05 ± 0.20 b	2.30 ± 0.06 c	0.83 ± 0.03 d	0.67 ± 0.03 d
N40	2.43 ± 0.09 a	1.73 ± 0.07 b	2.33 ± 0.07 a	1.13 ± 0.03 c	0.80 ± 0.06 c
N89	5.17 ± 0.62 a	3.40 ± 0.21 ab	2.87 ± 0.77 bc	0.73 ± 0.09 cd	0.60 ± 0.09 d

Note: Different letters indicate significant differences at the 0.05 level for the same strain under different PEG concentrations.

**Table 4:** Colony diameters of DSE and ERMF under drought stress (cm).

Strain	Cultured Alone	D79*N07	D65*N07	D79*N12	D65*N12
D79	5.00 ± 0.29 a	3.36 ± 0.19 b	-	4.43 ± 0.18 a	-
N12	3.23 ± 0.55 a	-	-	2.47 ± 0.22 b	1.67 ± 0.32 c
D65	3.53 ± 0.27 a	-	2.83 ± 0.22 ab	-	2.27 ± 0.15 b
D07	4.37 ± 0.5 a	2.03 ± 0.03 c	3.10 ± 0.31 b	-	-
Total inhibition rate%	-	86.35%	48.89%	34.90%	83.99%

Note: The lowercase letters indicate significant differences in the same strain under different confrontation combinations (*p* < 0.05). Each pair of strains will be placed in direct confrontation.

**Table 5:** Membership mean values and comprehensive evaluation scores of physiological parameters under drought stress.

Treatment	Drought	(X1)	(X2)	(X3)	(X4)	(X5)	(X6)	(X7)	Comprehensive Evaluation Score	Rank	Membership Mean	Total Rank
CK	T1	1.00	1.00	0.00	0.00	0.00	0.00	1.00	0.429	3	0.471	5
	T2	0.80	0.82	0.29	0.47	0.28	0.27	0.69	0.517	2		
	T3	0.50	0.29	1.00	1.00	0.35	1.00	0.28	0.631	1		
	T4	0.00	0.00	0.15	0.10	1.00	0.90	0.00	0.307	4		
D	T1	1.00	1.00	0.00	0.00	0.00	0.00	1.00	0.429	3	0.462	6
	T2	0.68	0.87	0.25	0.13	0.27	0.21	0.72	0.447	2		
	T3	0.07	0.38	1.00	1.00	0.45	0.95	0.49	0.620	1		
	T4	0.00	0.00	0.30	0.15	1.00	1.00	0.00	0.350	4		
N	T1	1.00	1.00	0.00	0.00	0.00	0.00	1.00	0.429	3	0.492	3
	T2	0.90	0.98	0.12	0.16	0.10	0.09	0.74	0.441	2		
	T3	0.77	0.34	1.00	1.00	0.42	0.85	0.49	0.696	1		
	T4	0.00	0.00	0.19	0.63	1.00	1.00	0.00	0.403	4		
D1N1	T1	1.00	1.00	0.00	0.00	0.00	0.00	1.00	0.429	3	0.513	2
	T2	0.81	0.67	0.18	0.30	0.20	0.16	0.71	0.433	2		
	T3	0.74	0.33	1.00	1.17	0.77	1.00	0.07	0.726	1		
	T4	0.00	0.00	0.27	1.00	1.00	0.97	0.00	0.463	4		
D1N2	T1	1.00	1.00	0.00	0.00	0.00	0.00	1.00	0.429	3	0.517	1
	T2	0.82	0.83	0.19	0.54	0.14	0.09	0.82	0.490	2		
	T3	0.65	0.44	1.00	1.00	0.78	1.22	0.17	0.751	1		
	T4	0.00	0.00	0.42	0.36	1.00	1.00	0.00	0.397	4		
D2N1	T1	1.00	1.00	0.00	0.00	0.00	0.00	1.00	0.429	3	0.475	4
	T2	0.87	0.82	0.23	0.07	0.15	0.16	0.77	0.439	2		
	T3	0.70	0.18	1.00	1.00	0.43	0.83	0.21	0.629	1		
	T4	0.00	0.00	0.40	0.41	1.00	1.00	0.00	0.401	4		

Note: X1 to X7 represent RWC, leaf electrolyte conductivity, SOD, soluble protein, soluble sugar, Pro, and MDA, respectively.

## Data Availability

The data that support the findings of this study are available from the corresponding author, WDL, upon reasonable request.

## Supplementary Materials

The following supporting information can be downloaded at: https://www.mdpi.com/article/10.3390/jof12050356/s1, Table S1:The expression of Metabolism.

## References

[1] Lum M.S., Hanafi M.M., Rafii Y.M., Akmar A.S.N. (2014). Effect of drought stress on growth, proline and antioxidant enzyme activities of upland rice. J. Anim. Plant Sci..

[2] Razi K., Muneer S. (2021). Drought stress-induced physiological mechanisms, signaling pathways and molecular response of chloroplasts in common vegetable crops. Crit. Rev. Biotechnol..

[3] Seki M., Umezawa T., Urano K. (2007). Regulatory metabolic networks in drought stress responses. Curr. Opin. Plant Biol..

[4] Aroca R., Bago A., Sutka M., Paz J.A., Cano C., Amodeo G., Ruiz-Lozano J.M. (2009). Expression analysis of the first arbuscular mycorrhizal fungi aquaporin described reveals concerted gene expression between salt-stressed and non-stressed mycelium. Mol. Plant Microbe Interact..

[5] Ahmadi A., Siosemardeh A. (2005). Investigation on the physiological basis of grain yield and drought resistance in wheat: Leaf photosynthetic rate, stomatal conductance, and non-stomatal limitations. Int. J. Agric. Biol..

[6] Azizi S., Kouchaksaraei M.T., Hadian J. (2021). Dual inoculations of arbuscular mycorrhizal fungi and plant growth-promoting rhizobacteria boost drought resistance and essential oil yield of common myrtle. For. Ecol. Manag..

[7] Shalaby O.A., Ramadan M.E. (2024). Mycorrhizal colonization and calcium spraying modulate physiological and antioxidant responses to improve pepper growth and yield under salinity stress. Rhizosphere.

[8] Shi Z., Mickan B., Feng G., Chen Y.L. (2015). Arbuscular mycorrhizal fungi improved plant growth and nutrient acquisition of desert ephemeral *Plantago minuta* under variable soil water conditions. J. Arid Land.

[9] Berthelot C., Perrin Y., Leyval C., Blaudez D. (2017). Melanization and ageing are not drawbacks for successful agro-transformation of dark septate endophytes. Fungal Biol..

[10] He C., Han T.T., Li X.N. (2022). Effects of Dark Septate Endophytes on the Performance and Soil Microbia of *Lycium ruthenicum* Under Drought Stress. Front. Plant Sci..

[11] Su H., Guo X., Gu L., Shi X.M., Zhou Y.Y., Wu F.L., Wang L. (2024). A novel growth-promoting dark septate endophytic fungus improved drought tolerance in blueberries by modulating phytohormones and non-structural carbohydrates. Tree Physiol..

[12] Harsonowati W., Baswarsiati, Nurrahma A.H.I., Iqbal R. (2026). Review on synergistic interactions of dark septate endophyte in promoting plant growth and drought resilience in the era of climate change. IOP Conf. Ser. Earth Environ. Sci..

[13] Ye Q., Li X., Long J., He X. (2024). Dark septate endophytes enhance the drought tolerance of *Haloxylon ammodendron* in sterilized and nonsterilized soil. Pedobiologia.

[14] Jia H., Geng Q., Li M., Wang R., Wang F., Deng Y., Xu W., Liu D. (2025). Dark septate endophytes promote the growth of *Cynodon dactylon* under drought stress and enhance its potential for use in the ecological restoration of slopes. Front. Plant Sci..

[15] Li X., Zhang X., Xu M.H. (2022). Improved Tolerance of *Artemisia ordosica* to Drought Stress via Dark Septate Endophyte (DSE) Symbiosis. J. Fungi.

[16] Zuo Y.L., Su F., He X.L., Li M. (2020). Colonization by dark septate endophytes improves the growth of *Hedysarum scoparium* under multiple inoculum levels. Symbiosis.

[17] Stevens P.F., Luteyn J., Oliver E.G.H., Bell T.L., Brown E.A., Crowden R.K., George A.S., Jordan G.J., Ladd P., Lemson K., Kubitzki K. (2004). Ericaceae. The Families and Genera of Flowering Plants.

[18] Perotto S., Daghino S., Martino E. (2018). Ericoid mycorrhizal fungi and their genomes: Another side to the mycorrhizal symbiosis?. New Phytol..

[19] Vohník M. (2020). Ericoid mycorrhizal symbiosis: Theoretical background and methods for its comprehensive investigation. Mycorrhiza.

[20] Mu D.Y., Du N., Zwiazek J.J. (2021). Inoculation with Ericoid Mycorrhizal Associations Alleviates Drought Stress in Lowland and Upland Velvetleaf Blueberry (*Vaccinium myrtilloides*) Seedlings. Plants.

[21] Fadaei S., Vaziriyeganeh M., Young M., Sherr I., Zwiazek J.J. (2020). Ericoid mycorrhizal fungi enhance salt tolerance in ericaceous plants. Mycorrhiza.

[22] Paltineanu C., Coman M., Nicolae S. (2018). Root system distribution of highbush blueberry crops of various ages in medium-textured soils. Erwerbs-Obstbau.

[23] Odokonyero K., Acuña T.B., Cardoso J.A., Rao I.M. (2016). Fungal endophyte association with *Brachiaria* grasses and its influence on plant water status, total non-structural carbohydrates and biomass production under drought stress. Plant Soil..

[24] Liu N., Jacquemyn H., Liu Q. (2022). Effects of a Dark Septate Fungal Endophyte on the Growth and Physiological Response of Seedlings to Drought in an Epiphytic Orchid. Front. Microbiol..

[25] Guo X.L., Wang Y.Q., Hu J.B., Wang D.L., Wang J.B., Shakeel M. (2023). Mixed inoculation of dark septate endophytic and ericoid mycorrhizal fungi in different proportions improves the growth and nutrient content of blueberry seedlings. Plant Biosyst..

[26] Guo X.L., Yuan L.F., Shakeel M. (2021). Screening of the plant growth-promoting mycorrhizal fungi in Guizhou blueberry. Rhizosphere.

[27] Gopal J., Iwama K. (2007). In vitro screening of potato against water-stress mediated through sorbitol and polyethylene glycol. Plant Cell Rep..

[28] Hussein E. (1957). Fungal Associations: II. Cultural studies on rhizoctonia solani Kühn, fusarium solani snyder and hansen, and other fungi, and their interactions. Ann. Bot..

[29] Chen G.M., Li S.P., Zhang H.H. (2009). Effects of mycorrhizal fungi associated with exogenous mycorrhizal fungi on their growth and neutral protease activity. J. Northwest A&F Univ. (Nat. Sci. Ed.).

[30] Yang Q., Meng P., Li J.Q., Zhang J.S., Guo L. (2010). Effects of soil water stress on photosynthesis and water use characteristics of *Eucommia ulmoides* leaves. Chin. Agrometeorol..

[31] Mahawar L., Kumar R., Shekhawat G.S. (2018). Evaluation of heme oxygenase 1 (HO1) in Cd and Ni induced cytotoxicity and crosstalk with ROS quenching enzymes in two to four leaf stage seedlings of *Vigna radiata*. Protoplasma.

[32] Dehghan M., Balouchi H., Yadavi A., Zare E. (2020). Improve wheat (*Triticum aestivum*) performance by brassinolide application under different irrigation regimes. S. Afr. J. Bot..

[33] Zhu S.P., Nong J.F., Luo G.T., Li Q.P., Wang F.H., Jing D. (2021). Varied tolerance and different responses of five citrus rootstocks to acid stress by principle component analysis and orthogonal analysis. Sci. Hortic..

[34] Roy R., Mostfa M.G., Wang J.X., Fornara D., Sarker T., Zhang R.Q. (2021). Revegetation intervention of drought-prone coal-mined spoils using *Caragana korshinskii* under variable water and nitrogen-phosphorus resources. Agric. Water Manag..

[35] Zhu H., Wang Y.X., Jiang J., Yang Z.Y., Li L.L., Yang H.Y. (2025). Metabolomic and physiological analysis of blueberry (*Vaccinium* spp.) in response to ericoid mycorrhizal fungi (*Oidiodendron maius* H14). Horticulturae.

[36] Shalaby O.A., Farag R., Ibrahim M.F.M. (2023). Effect of hydrogen sulfide and hydrogen peroxide on growth, yield and nutrient content of broccoli plants grown under saline conditions. Sci. Hortic..

[37] Smith S.E., Read D.J. (2008). Mycorrhizal Symbiosis (3rd Edition).

[38] Nxele X., Klein A., Ndimba B.K. (2017). Drought and salinity stress alters ROS accumulation, water retention, and osmolyte content in sorghum plants. S. Afr. J. Bot..

[39] Zhang Z., Zhang J., Xu G., Zhou L.W., Li Y.Q. (2019). Arbuscular mycorrhizal fungi improve the growth and drought tolerance of *Zenia insignis* seedlings under drought stress. New For..

[40] Chiappero J., Cappellari L.R., Alderete L.G.S. (2019). Plant growth promoting rhizobacteria improve the antioxidant status in Mentha piperita grown under drought stress leading to an enhancement of plant growth and total phenolic content. Ind. Crops Prod..

[41] Bernardo L., Carletti P., Badeck F.W., Fulvia Rizza F., Morcia C., Ghizzoni R., Rouphael Y., Colla G., Terzi V., Lucini L. (2019). Metabolomic responses triggered by arbuscular mycorrhiza enhance tolerance to water stress in wheat cultivars. Plant Physiol. Bioch..

[42] Xiong L., Zhu J.K. (2002). Molecular and genetic aspects of plant responses to osmotic stress. Plant Cell Environ..

[43] Ghosh U.K., Islam M.N., Siddiqui M.N., Cao X., Khan M.A.R. (2021). Proline, a multifaceted signalling molecule in plant responses to abiotic stress: Understanding the physiological mechanisms. Plant Biol..

[44] Tarnabi Z.M., Iranbakhsh A., Mehregan I., Ahmadvand R. (2020). Impact of arbuscular mycorrhizal fungi (AMF) on gene expression of some cell wall and membrane elements of wheat (*Triticum aestivum* L.) under water deficit using transcriptome analysis. Physiol. Mol. Biol. Plants.

[45] Sakamoto K., Ogiwara N., Kaji T. (2019). Transcriptome analysis of soybean (*Glycine max*) root genes differentially expressed in rhizobial, arbuscular mycorrhizal, and dual symbiosis. J. Plant. Res..

[46] Hemm M.R., Rider S.D., Ogas J., Murry D.J., Chapple C. (2004). Light induces phenylpropanoid metabolism in *Arabidopsis* roots. Plant J..

[47] Li X., He X.L., Hou L.F., Ren Y., Wang S.J., Su F. (2018). Dark septate endophytes isolated from a xerophyte plant promote the growth of *Ammopiptanthus mongolicus* under drought condition. Sci. Rep..

